# Systematized reporter assays reveal ZIC protein regulatory abilities are Subclass-specific and dependent upon transcription factor binding site context

**DOI:** 10.1038/s41598-020-69917-9

**Published:** 2020-08-04

**Authors:** Jehangir N. Ahmed, Koula E. M. Diamand, Helen M. Bellchambers, Ruth M. Arkell

**Affiliations:** 1 Early Mammalian Development Laboratory, John Curtin School of Medical Research, The Australian National University, Canberra, ACT 2601 Australia; 2 Present Address: Department of Pediatrics, Indiana University School of Medicine, Indianapolis, IN USA

**Keywords:** Transcription, Functional genomics, Mutagenesis, Genetic interaction, DNA-binding proteins, Transcription factors

## Abstract

The ZIC proteins are a family of transcription regulators with a well-defined zinc finger DNA-binding domain and there is evidence that they elicit functional DNA binding at a ZIC DNA binding site. Little is known, however, regarding domains within ZIC proteins that confer trans-activation or -repression. To address this question, a new cell-based trans-activation assay system suitable for ZIC proteins in HEK293T cells was constructed. This identified two previously unannotated evolutionarily conserved regions of ZIC3 that are necessary for trans-activation. These domains are found in all Subclass A ZIC proteins, but not in the Subclass B proteins. Additionally, the Subclass B proteins fail to elicit functional binding at a multimerised ZIC DNA binding site. All ZIC proteins, however, exhibit functional binding when the ZIC DNA binding site is embedded in a multiple transcription factor locus derived from ZIC target genes in the mouse genome. This ability is due to several domains, some of which are found in all ZIC proteins, that exhibit context dependent trans-activation or -repression activity. This knowledge is valuable for assessing the likely pathogenicity of variant ZIC proteins associated with human disorders and for determining factors that influence functional transcription factor binding.

## Introduction

The *Zic* genes encode a family of multi-functional transcription regulators required for a diverse range of biological processes in embryogenesis and adult homeostasis^1,2^. The defining feature of the corresponding proteins (ZIC1, ZIC2, ZIC3, ZIC4 and ZIC5) is a zinc finger domain (ZFD) composed of five tandem Cys2His2 (C2H2)-type zinc fingers (ZFs). The ZFD is most closely related to the GLI, GLIS and NKL families but the ZIC ZFD is distinguished by an atypical first ZF. Generally, one to five amino acids separate the two cysteines of a C2H2 ZF but, in the first ZF of ZIC proteins in species so far examined, this number ranges from 6 to 38^3^. In addition, the first two ZFs of ZIC proteins contain a tryptophan residue between the canonical cysteines and structural analysis suggests these two ZFs may form a single structural unit called a CWCH2 motif^3,4^. The ZIC ZFD was one of the first shown to participate in both DNA binding and protein binding^5^ and this dual capability contributes to the myriad molecular roles of ZIC proteins (reviewed in^6^). Outside of the ZFD, phylogenetic analysis has identified two other regions conserved amongst ZIC proteins. The N-terminal ZOC box is found within ZICs 1, 2 and 3^3^ and participates in protein–protein interactions^7,8^. Just N-terminal of the ZFD there is another short, highly conserved sequence, called the ZFNC, which is of unknown function. Each of the ZIC proteins also contain low complexity regions, including stretches of Alanine (ZICs 1, 2, 3, 4 and 5), Histidine (ZICs 1, 2 and 3), as well as Serine/Glycine (ZICs 2 and 5) and Proline (ZIC5)^2^.

On the basis of protein sequence conservation, ZIC proteins are classified into two Subclasses: Subclass A proteins (ZICs 1, 2 and 3) share a conserved ZFD and contain the N-terminal ZOC box, whereas the Subclass B proteins (ZIC4 and 5) have a divergent first ZF and lack the ZOC box. The Subclass division has its basis in invertebrate evolution where a single ancestral *Zic* gene underwent tandem duplication and divergence prior to the genome duplications early in the vertebrate lineage^2,3^. Many vertebrate *Zic* genes therefore still exist as divergently transcribed, tandem pairs. For example, in the teleost lineage *zic1* and *zic4* form a gene pair as do *zic2*/*zic5* and *zic3*/*zic6*^9^. The *zic6* gene has been lost from tetrapods and the precursor to the mammalian X-chromosome underwent a deletion/inversion event such that *zic6* was lost and the orientation of the *zic3* gene was reversed. Consequently, in mammals ZIC1/ZIC4 and ZIC2/ZIC5 remain as gene pairs whereas ZIC3 is an X-linked singleton. A corollary of the gene-pair arrangement is that *Zic1* and *Zic4* as well as *Zic2* and *Zic5* have extensive overlap in expression domains in multiple organisms^9–13^. Exactly how these sequence differences influence the function of the distinct Subclass proteins is unknown.

A significant impediment to understanding the sequence/function relationships for ZIC proteins is the shortage of robust cell-based reporter assays. Such assays have been difficult to generate because ZIC proteins produce high background in the reporter assays tested to date; an effect attributed to their ability to stimulate a range of widely used basal promoters (such as TK and SV40). ZIC expression can therefore lead to false positive effects at reporter constructs driven by these promoters^14^ and can cross-regulate plasmids designed to normalise for transfection efficiency differences^15^. Other features contributing to the dearth of ZIC specific cell-based assays are the poor specificity and/or strength of commercially available antibodies,as well as the lack of non-DNA interacting ZIC variant proteins and in vitro validated DNA binding elements (or ZIC response elements,ZREs). Initial attempts to identify a consensus ZIC DNA binding site used yeast one-hybrid^16,17^ or cDNA selection^14^ approaches, one of which identified ZIC protein binding sites on the *APOE* promoter. This element remains the most commonly employed promoter for ZIC trans-activation assays^15,17,18^. There is however no evidence that *APOE* is an in vivo ZIC target and during early murine embryonic development, when ZIC proteins are most active, the *Apoe* gene and *Zic* genes are not co-expressed^12,19^. More recent experiments^20,21^ identify a different ZIC binding sequence to that identified at the *APOE* promoter or in previous experiments^14,16^. The consensus ZIC3 DNA-binding motif identified independently by both protein binding microarray^20^ and ChIP-chip experiments^21^ is CC^C^/_T_GCTGGG. Murine ZIC3 binds this site at the *Nanog* proximal promoter and directs expression of *Nanog*^21^, a pluripotency gene that is co-expressed with *Zic3* during early murine development^12,22^. In this context, ZIC3 exhibits functional DNA binding, i.e. ZIC3 both binds DNA and elicits a transcriptional response.

Here we have addressed issues associated with cell-based ZIC reporter assays by constructing a suite of reporter and expression constructs optimised for interrogating the trans-activation ability of ZIC proteins in HEK293T cells. A vector backbone and basal promoters refractory to ZIC activation were identified and it was shown that ZREs are not subjected to high stimulation by endogenous TFs in HEK293T cells. The ZIC compatible vector was used to build reporter constructs that contain either the multimerised ZIC3 site identified in Badis, et al.^20^ and Lim, et al.^21^ or recently identified ZIC-specific genomic targets. Additionally, a suite of ZIC expression constructs (each with an N-terminal epitope tag for co-ordinated detection) were built and a variant of each ZIC protein generated that cannot interact with reporter DNA. This systematized group of reporter and expression constructs enable comparison between ZIC family members and test ZREs in a ‘one variable at a time’ manner. This system demonstrates for the first time that trans-activational abilities of the two ZIC Subclasses are different. The Subclass A proteins drive high levels of transcription at the identified ZIC consensus binding site in isolation, whereas the Subclass B proteins do not. The trans-activation ability of ZIC3 maps to two newly identified domains that are evolutionarily conserved amongst Subclass A, but not Subclass B ZICs. When, however, the ZIC binding site is co-located with other transcription factor binding sites (TFBS) in a multiple transcription factor locus (MTL), all ZIC proteins are able to stimulate transcription, and this ability requires ZIC/DNA interaction. In addition to the trans-activation domains, the ZIC3 protein contains several domains, some of which are found amongst all ZIC proteins, which act in a context dependent manner to regulate transcription. The activity of a ZIC protein at a ZRE is therefore determined by its particular complement of transcriptional regulatory domains and the co-location of other TFBS at the genomic locus.

## Results

### Wildtype and non-reporter-DNA-interacting variant ZIC proteins

**Figure 1:**
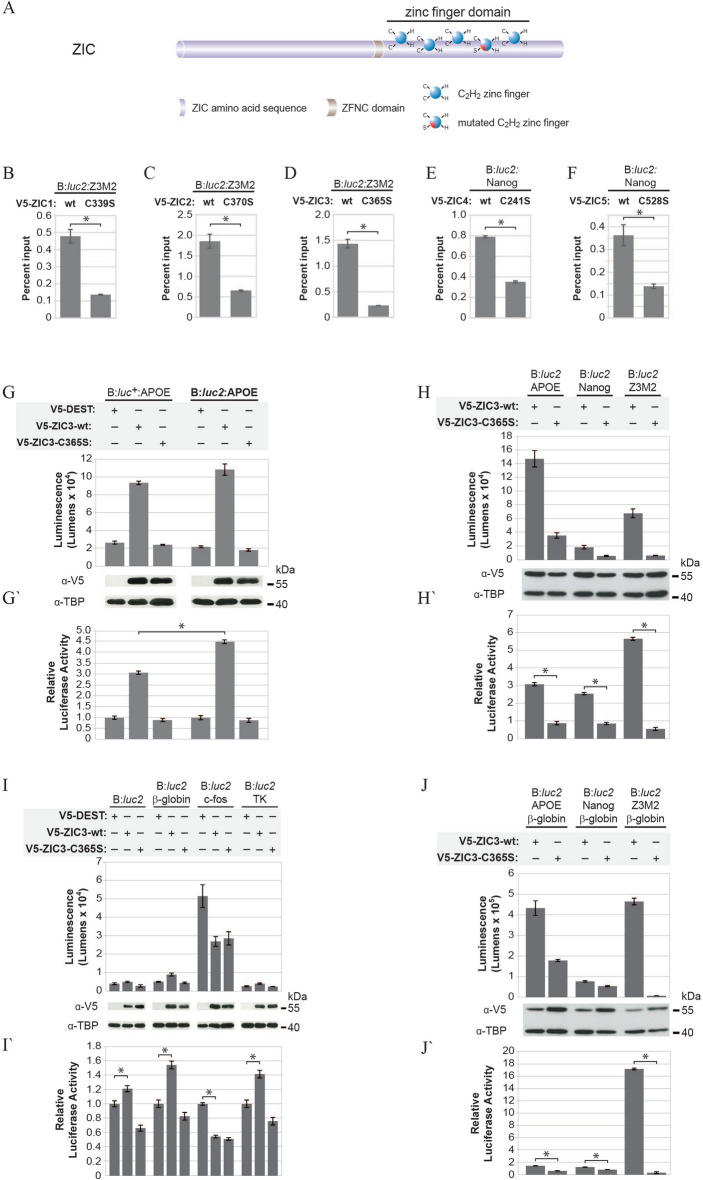
A new trans-activation assay for ZIC proteins. (**A**) Schematic representation of the missense mutation [second cysteine (C) converted to serine (S)] introduced into each ZIC protein. (**B**–**F**) qPCR (N = 3) output following pIP from HEK293T cells co-transfected with the reporter construct (above line) and expression constructs (below line) shown. Error bars represent SD between three repeats, *p < 0.01 t-Test. (**G**–**J**) HEK293T cells were co-transfected with the reporter constructs (above line) and expression constructs (below line) shown. V5-DEST was also co-transfected with each reporter construct to measure background (not shown in panels **H** and **J,** see Fig. S1 for all data). For each transfection, luminescence was measured 24 h post-transfection in each of three replicate samples and each transfection repeated three times (N = 3). In each panel, the top graph (**G**–**J**) shows one representative experiment with the corresponding western blot. Error bars represent the SD between the three replicates. Expression of transfected proteins was confirmed with α-V5 and the α-TBP blot served as nuclear fraction loading control. Although cropped blots were used, the gels were run under the same experimental conditions. The bottom graph (**G′**–**J′**) shows the mean RLA (normalised to V5-DEST such that the V5-DEST value becomes 1; not shown in **H′** and **J′**) value from three independent repeats, Error bars represent SEM. *p < 0.01 ANOVA.

To generate wildtype ZIC proteins, a cDNA corresponding to each human ZIC protein was cloned into the same vector (V5-DEST) to enable production of N-terminally V5 epitope tagged proteins following transfection in mammalian cell lines. The use of the same vector backbone and an epitope tag facilitates meaningful comparison of different ZIC proteins in transfections, reporter assays and western blot/antibody hybridisation procedures. In reporter assays, signal is generated when the overexpressed protein binds a test DNA element contained within the reporter construct and elicits transcription. Several non-specific activities can confound interpretation of these assays, for example, luciferase activity could be stimulated by (non ZIC) endogenous transcription factors acting on cryptic DNA binding sites in the plasmid. To distinguish between these possibilities, negative control ZIC protein variants that do not interact with the reporter plasmid DNA are required. Since a missense mutation affecting a key cysteine residue in the 4th ZF of ZIC2, that causes the C370S amino acid substitution, eliminates DNA binding ability^15^, an orthologous substitution was made in cDNAs encoding each of the four remaining human ZIC proteins (Fig. 1A). These were tested by plasmid-IP-qPCR (pIP; a modified version of ChIP-qPCR using plasmid DNA) following co-transfection of expression plasmids and reporter constructs containing a known ZRE. Each expression vector (wildtype and mutant) was also co-transfected with the negative control empty parent reporter construct (pGL4.20 designated B:*Luc2*). Each ZIC mutant displayed significantly reduced enrichment at the ZRE relative to the corresponding wildtype protein (Fig. 1B–F). In all cases (ZIC1-5) the ZIC mutant protein reduced ZRE interaction to levels less than or equivalent to that shown with the negative control plasmid (data not shown) confirming that in each case this mutation prevents interaction between the ZIC protein and the reporter DNA.

### A robust and sensitive ZIC3 transcription assay

The reporter construct most often used in cell-based ZIC trans-activation assays consists of a genomic fragment from the human *APOE* promoter (− 189/+ 1) cloned into the promoterless pXP2 vector containing the *Photinus pyralis* luciferase cDNA^17^, referred to here as B:*luc*^+^:APOE. In contrast to pXP2, a range of new generation vectors have been developed in which cryptic DNA binding sites for endogenous transcription factors are minimised and the luciferase cDNA codon-optimized for expression in mammalian systems^23^. To establish a trans-activation assay suitable for dissecting ZIC protein function, the *APOE* promoter fragment was transferred to a new generation vector backbone pGL4.20 (B:*luc2*) producing B:*luc2*:APOE. For direct comparison, the B:*luc*^+^:APOE and B:*luc2*:APOE reporter constructs were transfected into HEK293T cells with either an empty expression vector (V5-DEST), V5-tagged wildtype ZIC3 (V5-ZIC3-wt) or the V5-tagged non-DNA-interacting form of ZIC3 (V5-ZIC3-C365S). Although the B:*luc2*:APOE displayed similar background levels to B:*luc*^+^:APOE (i.e. in the presence of V5-DEST), there was a significant increase in ZIC3 dependent luciferase activity from B:*luc2*:APOE in comparison to B:*luc*^+^:APOE. Importantly, this difference could be clearly seen when data was expressed relative to the luciferase activity in the presence of V5-DEST (to calculate the relative luciferase activity) (Fig. 1G,G′). For both reporter constructs, expression of ZIC3-C365S reduced luciferase activity to background levels, indicating the increase in luciferase activity in the presence of ZIC3-wt is dependent on interaction with the reporter DNA.

To identify suitable ZREs three sequences were tested: (1) the *APOE* promoter fragment^17^, (2) the *Nanog* proximal promoter element^21^ and, (3) a multimerised (containing six tandem copies) ZIC binding sequence (designated Z3M2) corresponding to that identified by both protein binding microarray^20^ and ChIP-chip experiments^21^. Each sequence was cloned upstream of the *luc2* cDNA in B:*luc2* to generate B:*luc2*:APOE, B:*luc2*:Nanog and B:*luc2*:Z3M2, respectively. When transfected into HEK293T cells in the presence of V5-ZIC3-wt or V5-ZIC3-C365S each reporter construct was trans-activated by ZIC3 in a DNA-interaction dependent manner (Figs. 1H, S1A) with the *APOE* promoter fragment driving the highest luciferase activity. When data was expressed relative to the luciferase activity at each reporter in the presence of V5-DEST (Fig. 1H′) it became apparent that not all signal derived from the *APOE* promoter fragment was ZIC3 dependent and that other endogenous transcription factors contributed to the high luciferase activity in this experiment. In contrast ZIC3 made a significant contribution to the activity at both the *Nanog* and Z3M2 elements, where the Z3M2 element led to high enrichment over the background trans-activation level.

**Figure 2:**
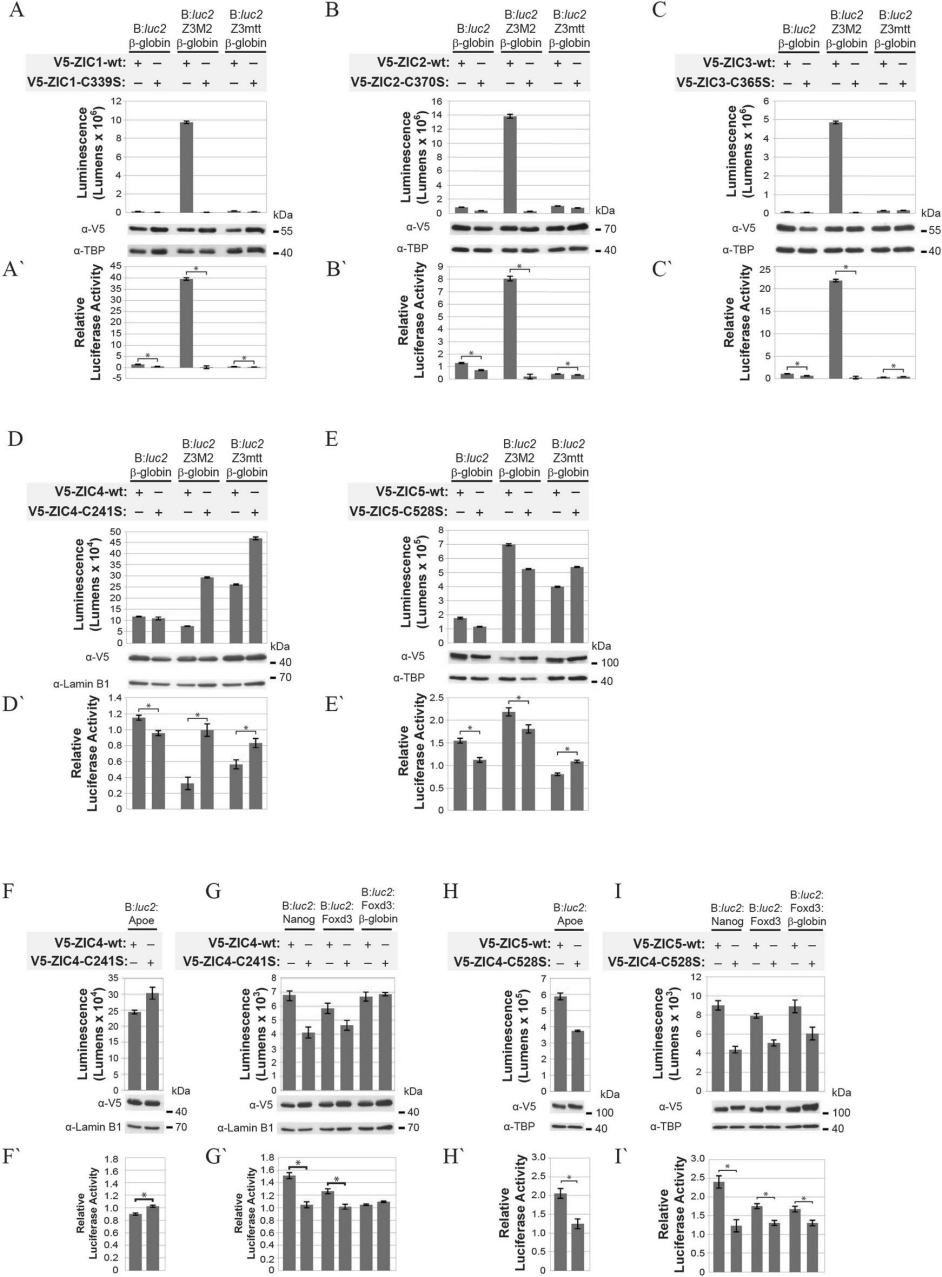
Trans-activation spectrum of ZIC proteins. HEK293T cells were co-transfected with the reporter constructs (above line) and expression constructs (below line) shown. V5-DEST was also co-transfected with each reporter to measure background (not shown, see Fig. S2 for all data). For each transfection, luminescence was measured 24 h post-transfection in each of three replicate samples and each transfection repeated three times (N = 3). In each panel, the top graph (**A–I**) shows one representative experiment with the corresponding western blot. Error bars represent the SD between the three replicates. Expression of transfected proteins was confirmed with α-V5 and the α-TBP or α-Lamin B1 blot served as nuclear fraction loading control. Although cropped blots were used, the gels were run under the same experimental conditions. The bottom graph (**A′**–**I′**) shows the mean RLA value (normalised to V5-DEST such that the V5-DEST value becomes 1; note the normalised V5-DEST is not shown) calculated from three independent repeats. Error bars represent SEM. *p < 0.01 ANOVA.

In previous studies the ZIC proteins have been shown to stimulate basal promoters commonly used in heterologous expression systems (such as TK and SV40)^14^ or ZIC protein variants interacted unpredictably with co-transfected control plasmids (with TK as promoter)^15^. In HEK293T cells, the SV40 promoter is strongly stimulated by ZIC proteins (up to 25 fold), whereas the TK promoter led to 4–8-fold stimulation^14^. In our experiments ZIC3 weakly stimulated the TK promoter (1.4 fold increase) (Fig. 1I,I′), suggesting that previous observations involving the TK promoter may be due to other vector elements in the pGL2 vector (in comparison to the pGL4 vector backbone used here). Two additional promoters (β-globin and c-fos) were tested for trans-activation via ZIC3. The β-globin promoter was weakly stimulated while transcription was repressed at the c-fos promoter (Fig. 1I,I′). Overall these studies indicated that some basal promoters were compatible with ZIC trans-activation studies using the B:*luc2* vector in HEK293T cells. To further improve trans-activation via ZREs, a basal promoter was included in the reporter construct. The β-globin promoter increased luciferase activity when combined with the synthetic Z3M2 enhancer (Figs. 1J,J′, S1B) (unlike the TK and c-fos promoters; see Supplementary Fig. S1C–E) and did not react unpredictably with ZIC mutants (Fig. 2A–E). Inclusion of the β-globin promoter with the genomic promoters (B:*luc2*:APOE:β-globin and B:*luc2*:Nanog:β-globin) did not, however, further elevate trans-activation (Figs. 1J,J′, S1B).

### The Subclass A and B ZIC proteins have different trans-activation abilities

The DNA binding site sequence used in the Z3M2 construct was identified either in a ZIC3 specific experiment^21^, or in a Subclass A experiment (i.e. with ZIC1, 2 and 3 proteins)^20^. The ability of each ZIC protein to trans-activate this motif was examined in HEK293T cells. All Subclass A ZIC proteins produced high level, DNA interaction dependent trans-activation of the B:*luc2*:Z3M2:β-globin construct (Figs. 2A–C′, S2). To further confirm that protein binding at the ZIC3 DNA binding site sequence was responsible for the luciferase activity, the site was mutated in all six tandem copies (construct B:*luc2*:Z3mtt:β-globin), reducing luciferase activity by wildtype ZIC1, 2 or 3 to that of the respective non-DNA-interacting mutants. In contrast, the Subclass B ZIC proteins (ZIC4 and 5) drove very little luciferase activity from the Z3M2 element (Figs. 2D–E′, S2). In the case of ZIC4, luciferase activity was reduced in the presence of the wildtype protein (relative to that of the B:*luc2*:β-globin construct) and this effect was dependent upon interaction with DNA at the Z3M2 motif. The ZIC5 protein had a mild stimulatory effect on luciferase activity at the Z3M2 element which was not entirely eliminated by an inability to interact with the reporter, although mutation of the Z3M2 site prevented ZIC5 dependent trans-activation suggesting that some of the luciferase activity seen in the presence of wildtype ZIC5 is due to endogenous transcription factors. Overall the data suggest that the Subclass B ZIC proteins do not exhibit functional binding (i.e. binding which results in transcriptional activation) at the ZIC3 motif in isolation.

**Figure 3:**
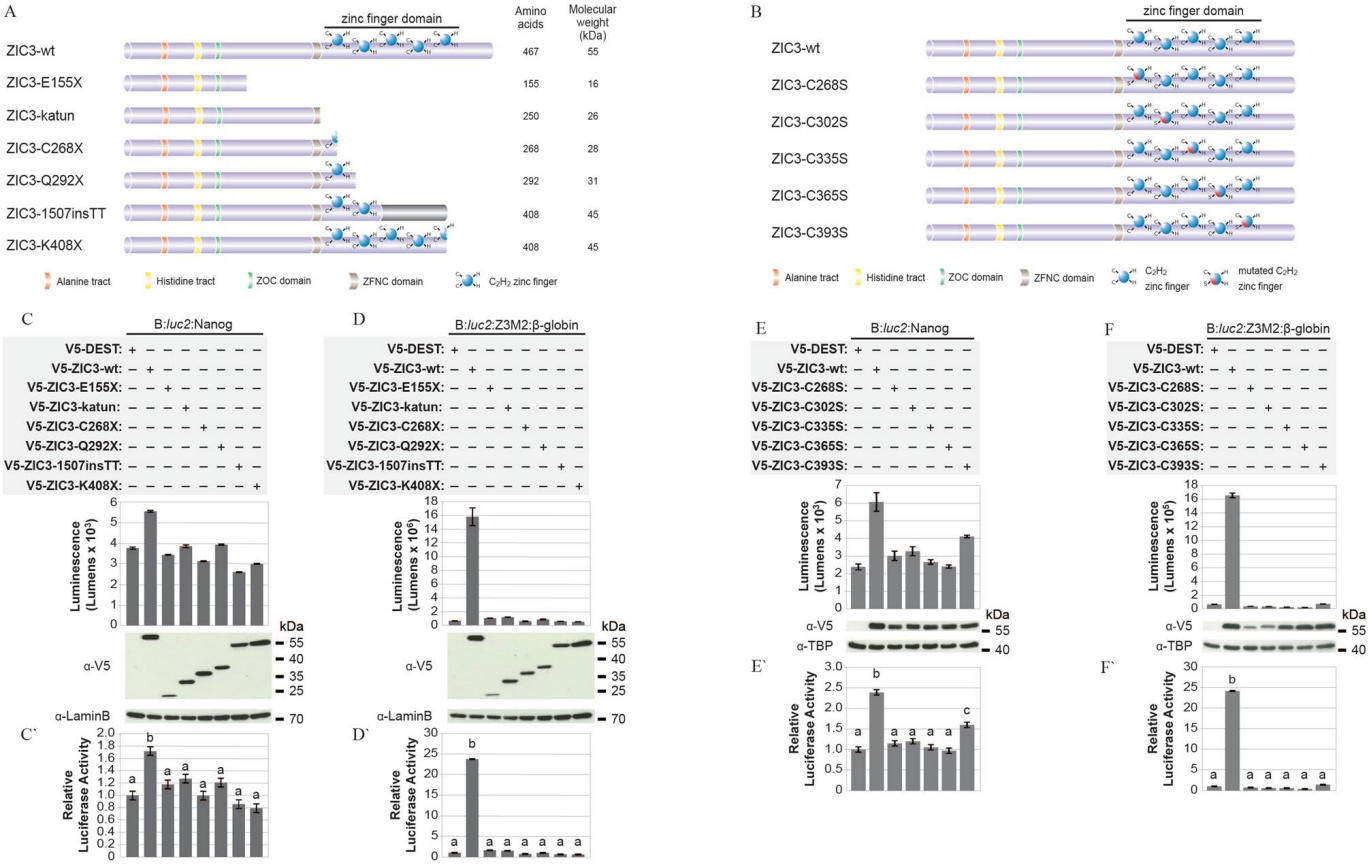
C-terminal regions and ZFD of ZIC3 are required for trans-activation. (**A**,** B**) Schematic representation of wildtype ZIC3 and (**A**) PTC-containing mutants or (**B**) ZF mutants containing a missense mutation in the second cysteine (C) [converted to serine (S)]. (**C**–**F**) HEK293T cells were co-transfected with the reporter construct (above line) and expression constructs (below line) shown. For each transfection, luminescence was measured 24 h post-transfection in each of three replicate samples and each transfection repeated three times (N = 3). In each panel, the top graph (**C**–**F**) shows one representative experiment with the corresponding western blot. Error bars represent SD between the three replicates. Expression of the transfected proteins was confirmed with α-V5 and the α-LaminB1 (**C**, **D**) or α-TBP (**E**, **F**) blots served as nuclear fraction loading control. Although cropped blots were used, the gels were run under the same experimental conditions. The bottom graph (**C′**–**F′**) shows mean RLA (normalised to V5-DEST such that the V5-DEST value becomes 1), N = 3. Error bars represent SEM. a, b and c: *p* < 0.01 ANOVA.

**Figure 4:**
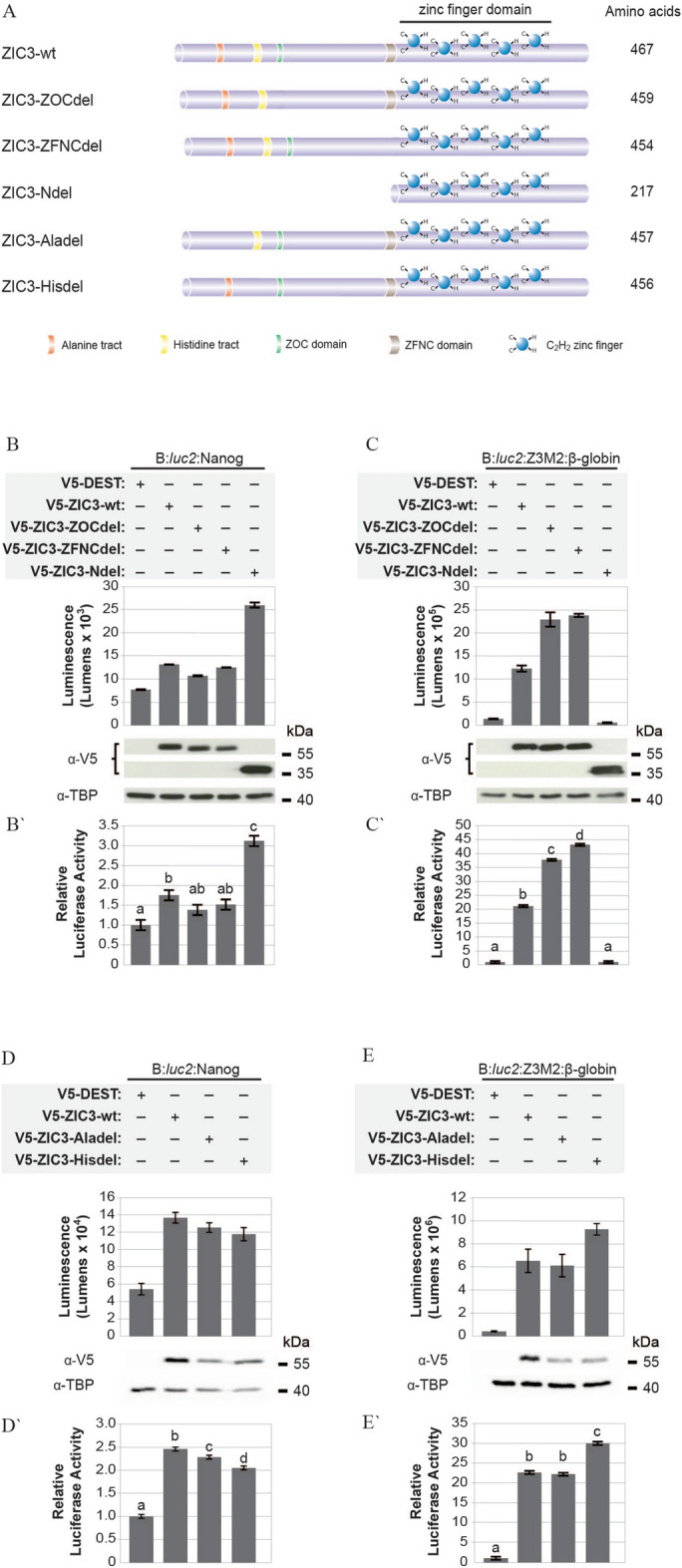
N-terminal of ZIC3 is required for trans-activation. (**A**) Schematic representation of the ZIC3 deletion mutants used. ZIC3-ZOCdel is missing the ZOC domain (green band); ZIC3-ZFNCdel is missing the ZFNC domain (brown band); ZIC3-Ndel is missing the entire N-terminal (amino acids preceding ZF1); ZIC3-Aladel is missing the Alanine tract (orange band); and ZIC3-Hisdel is missing the Histidine tract (yellow band). (**B**–**E**) HEK293T cells were co-transfected with the reporter construct (above line) and expression constructs (below line) shown. For each transfection, luminescence was measured 24 h post-transfection in each of three replicate samples and each transfection repeated three times (N = 3). In each panel, the top graph (**B**–**E**) shows one representative experiment with the corresponding western blot. Error bars represent SD between the three replicates. Expression of the transfected proteins was confirmed with α-V5 and the α-TBP blot served as nuclear fraction loading control. Although cropped blots were used, the gels were run under the same experimental conditions. The bottom graph (**B′**–**E′**) shows mean RLA (normalised to V5-DEST such that the V5-DEST value becomes 1), N = 3. Error bars represent SEM. a, b, c and d: *p* < 0.01 ANOVA.

**Figure 5:**
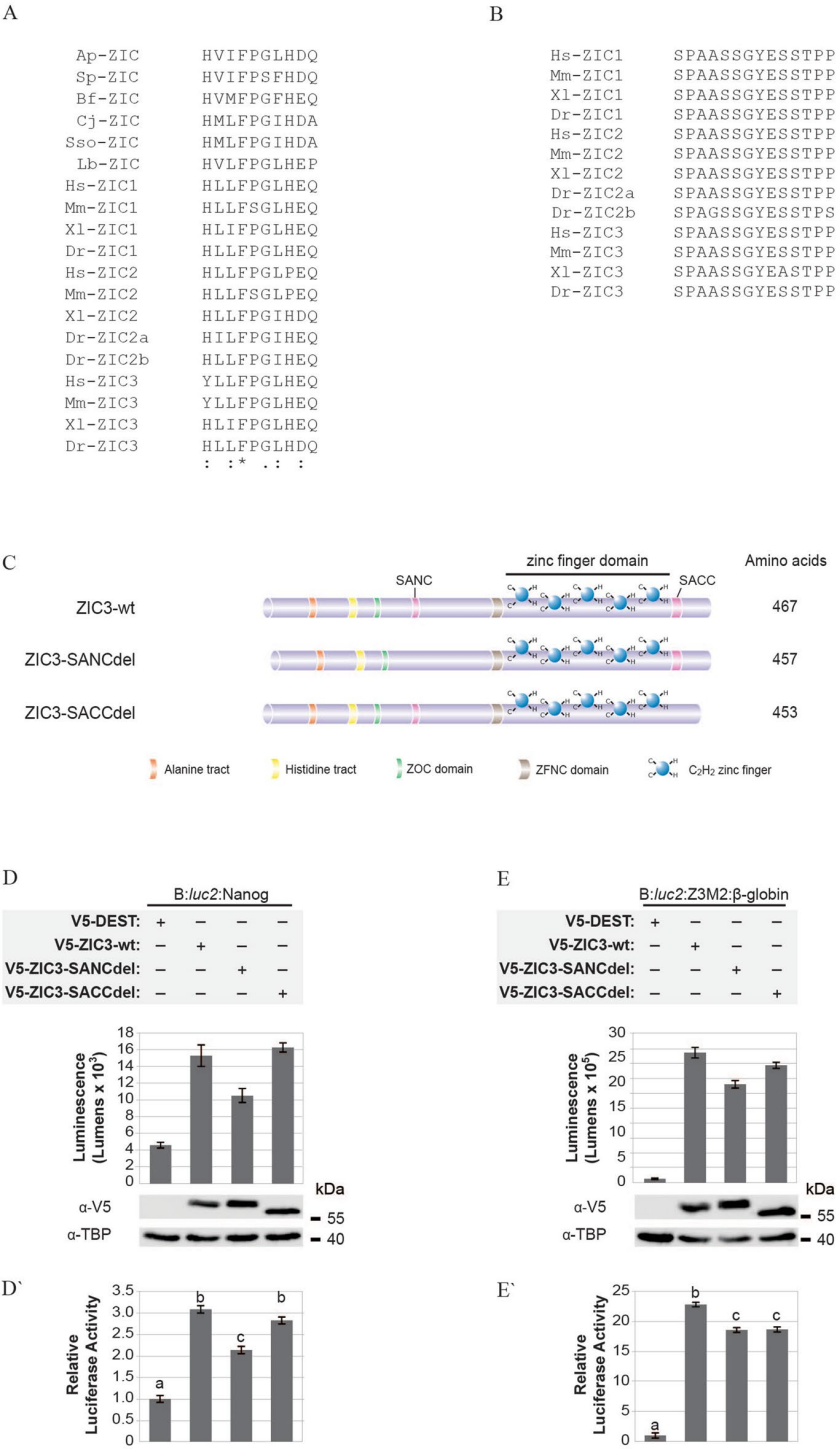
Subclass A ZIC proteins contain two evolutionary conserved regions that regulate transcription. (**A**, **B**) ZIC protein sequence alignment of (**A**) the N-terminal (SANC) and (**B**) the C-terminal (SACC) evolutionary conserved regions found in a variety of metazoan species: *Ap*, *Asterina pectinifera*; *Sp*, *Strongylocentrotus purpuratus*; *Bf*, *Branchiostoma floridae*; *Cj*, *Corbicula* sp.; *Sso*, *Spisula solidissima*; *Lb*, *Loligo bleekeri*; *Hs*, *Homo sapiens*; *Mm*, *Mus musculus*; *Xl*, *Xenopus laevis*; *Dr*, *Danio rerio*. Asterick (*) = identical residues. Colon (:) = functionally conserved residues. Period (.) = weak conservation of residue. (**C**) Schematic representation of the ZIC3 deletion mutants created. ZIC3-SANCdel is missing the SANC domain; ZIC3-SACCdel is missing the SACC domain. (**D**, **E**) HEK293T cells were co-transfected with the reporter construct (above line) and expression constructs (below line) shown. For each transfection, luminescence was measured 24 h post-transfection in each of three replicate samples and each transfection repeated three times (N = 3). In each panel, the top graph (**D**, **E**) shows one representative experiment with the corresponding western blot. Error bars represent SD between three internal replicates. Expression of the transfected proteins was confirmed with α-V5 and the α-TBP blot served as nuclear fraction loading control. Although cropped blots were used, the gels were run under the same experimental conditions. The bottom graph (**D′**, **E′**) shows mean RLA (normalised to V5-DEST such that the V5-DEST value becomes 1). Error bars represent SEM. a, b, and c: *p* < 0.01 ANOVA.

To examine whether ZIC4 and ZIC5 stimulate transcription from genomic elements a suite of reporter constructs, each containing a different MTL, were assayed. Along with B:*luc2*:APOE and B:*luc2*:Nanog constructs, a ZRE from the murine *Foxd3* gene was also cloned into B:*luc2* without or with the β-globin promoter to generate B:*luc2*:Foxd3 and B:*luc2*:Foxd3:β-globin. Upon co-transfection in HEK293T cells of each reporter with ZIC4 wildtype or its non-DNA-interacting variant, ZIC4 displayed DNA-interacting dependent trans-activation ability at the *Nanog* and *Foxd3* elements (Figs. 2G,G′, S2) that was within range of the activity displayed by the subclass A ZIC3 protein at the B:*luc2*:Nanog construct (Figs. 3, 4, 5). The *APOE* construct again showed non-specific activity and the β-globin promoter did not further enhance trans-activation at the *Foxd3* element (Figs. 2F–G′, S2). The presence of ZIC5 led to similar results, although the fold induction via each element was larger than that for ZIC4 and the trans-activation at the *APOE* element was dependent upon ZIC5 interaction with DNA (Figs. 2H–I′, S2). Overall it appears that ZIC4 and ZIC5 can act as context dependent transcription factors at MTLs and that the binding of other transcription factors at flanking DNA facilitates ZIC4 and ZIC5 trans-activation ability.

### The C-terminus is required for ZIC3 trans-activation ability

To determine which regions of the ZIC3 protein contribute to its trans-activation ability a series of truncated proteins were assayed. The protein truncations (Fig. 3A) were designed based on premature termination codon (PTC) containing *ZIC3* variants associated with Heterotaxy in humans^24,25^ or mice^18^ and each was missing the C-terminus and either the whole ZFD (ZIC3-E155X and ZIC3-katun) or parts of it (ZIC3-C268X, ZIC3-Q292X, ZIC3-1507insTT, ZIC3-K408X). Each protein was stably expressed upon transfection of the corresponding plasmid into HEK293T cells (see WBs in Fig. 3C,D) and each was able to accumulate within the nucleus, although not always to the same degree as the wildtype protein, as assessed by immunofluorescence (see Supplementary Figs. S3 and S4). The ability of each protein to trans-activate a ZRE was assessed using the multimerised ZIC3 binding site (B:*luc2*:Z3M2:β-globin) or the *Nanog* MTL (B:*luc2*:Nanog). Each protein was unable to trans-activate either reporter (Fig. 3C–D′), further confirming the importance of the ZFD and C-terminus.

The most complete of the tested proteins (ZIC3-K408X) truncates at amino acid 408 which lies between the two canonical histidines in ZF5 of ZIC3. To distinguish if the inability to trans-activate reporter sequences by this protein is due to a defect in ZF5 or lack of the C-terminal domain, ZIC3 proteins with a mutation at the canonical cysteine in each ZF were produced (Fig. 3B) and tested in the same assays. Each of these proteins (ZIC3-C268S, ZIC3-C302S, ZIC3-C335S, ZIC3-C365S and ZIC3-C393S) is stably expressed in HEK293T cells and localises to the nucleus (see Supplementary Figs. S3 and S4). Mutation of ZF1-4 ablates trans-activation ability, whereas mutation of ZF5 does not fully eliminate trans-activation ability (Fig. 3E–F′). The contrast between the partial loss of trans-activation by the ZF5 mutant and the total loss shown by ZIC3-K408X indicates that C-terminal sequences necessary for trans-activation are located downstream of the ZFD.

### Multiple N-terminal domains contribute to the control of ZIC-dependent transcription

To examine the effect of the N-terminus, a series of deletion mutants were created (Fig. 4A). Each of these proteins was stably expressed in HEK293T cells and localised to the nucleus (see Supplementary Figs. S3 and S4). The N-terminal deletion (ZIC3-Ndel) was incapable of eliciting transcription from the multimerised ZIC3 binding site but drove vast over-stimulation of the *Nanog* promoter relative to wildtype ZIC3 protein (Fig. 4B–C′). This suggests an interaction with other DNA bound proteins at the *Nanog* promoter enables trans-activation via ZIC3-Ndel and in this context the N-terminus has a strong transcriptional repression activity. Specific deletion of the N-terminus conserved domains (ZOC and ZFNC) and the low complexity Histidine repeat region (Hisdel) also gave contrasting results at the multimerised and MTL reporter constructs. In each case deletion of these domains led to hyper-stimulation of the isolated ZIC3 binding site but either had no effect on or decreased transcription at the *Nanog* promoter (Fig. 4B–E′). Deletion of the low complexity Alanine repeat region (Aladel) did not alter trans-activation from the multimerised ZIC3 binding site, but caused a mild reduction in transcription at the *Nanog* promoter. Overall several regions within the N-terminus (the ZOC and ZFNC domains as well as the Alanine and Histidine repeats) each appear to be necessary for optimal trans-activation at the *Nanog* promoter, whereas three of these domains (ZOC, ZFNC and Histidine repeat) act as repressive domains at the multimerised ZIC3 binding site.

### Subclass A-specific evolutionary conserved domains contribute to trans-activation

To identify any other regions of the ZIC3 protein that may contribute to transcriptional control, we conducted a two-pronged search to find: (1) additional evolutionary conserved regions within the amino acid sequence of all ZIC proteins, and (2) regions enriched in amino-acids known to be over-represented in trans-activation domains, such as acidic [aspartic acid (D) or glutamic acid (E)], proline (P), glutamine (Q), or serine (S) and threonine (T) residues^26^. This identified two previously unannotated, evolutionary conserved domains within the Subclass A ZIC proteins: (1) a ten amino acid sequence in the N-terminal, referred to here as SANC (Subclass A N-terminal Conserved) (Fig. 5A) and since identified as Conserved Domain 3 (CD3) in a thorough search of 121 ZIC protein sequences from 22 animal phyla^27^, and (2) a less-deeply conserved 14 amino acid sequence (nine of which are known to be present in trans-activation domains) in the C-terminus, referred to here as SACC (Subclass A C-terminal Conserved) (Fig. 5B). The influence of these regions on the trans-activation ability of ZIC3 was assessed by interstitial deletion of the respective domains (Fig. 5C). The resulting V5-tagged proteins were stably expressed and localised in the nucleus (see Supplementary Figs. S3 and S4). Both domains were necessary for ZIC3 stimulated trans-activation either at the *Nanog* promoter and multimerised site (SANC) or only at the multimerised site (SACC) (Fig. 5D–E′).

## Discussion

The ZIC family of transcription factors are essential for multiple lineage decisions during embryonic development and recent work suggests they play important roles in stem cell function^21,28–30^ and their dysregulations leads to cancer^31–36^. Despite numerous roles in embryonic development and adult homeostasis our knowledge regarding protein features that enable ZIC functional diversity is incomplete. In vitro reporter assays remain an important mechanism for identifying functional domains within transcription factors, for assessing likely pathogenicity of variant proteins and for determining factors that influence functional transcription factor binding^37,38^. Such comparisons rely on the ability to examine one variable at a time. Here, a systematized suite of vectors, in which each expression plasmid and each reporter plasmid has an identical backbone and in which reporter plasmids utilise promoters that are refractory to ZIC protein influence in the cell line used, was built. This allowed meaningful comparisons of ZIC transcription factor activity across wildtype or variant family members and between synthetic and genomic target DNA sequences. A deletion strategy identified regions necessary for functional DNA binding in HEK293T cells and demonstrated that the trans-activation activity of the ZIC proteins is modular and context dependent. In ZIC3, the trans-activation activity is enhanced by two Subclass A-specific, evolutionarily conserved domains (SANC and SACC). The Subclass B proteins lack these trans-activation domains and do not produce functional binding at an isolated, but multimerised, ZIC binding site. For a further set of domains (the ZOC, ZFNC and low complexity regions), their contribution to ZIC3 trans-activation ability is context dependent. For example, a domain may decrease trans-activation at the isolated ZIC3 DNA binding site but promote transcription when the DNA binding site is embedded within a genomic fragment that contains multiple transcription factor binding sites (a MTL). The overall ability of each ZIC protein to elicit functional binding at a given ZRE is therefore dependent upon both its complement of transcriptional control domains and the DNA sequence surrounding the ZRE.

Two complementary studies of ZIC Subclass A binding (a ChIP-chip murine ZIC3 experiment and a protein binding microarray^20,21^ conducted with the DNA binding domain of ZIC1, ZIC2 and ZIC3) previously identified highly similar binding sequences for these proteins. The motif proposed by Lim et al. (CC^C^/_T_GCTG_G) is the reverse complement of the secondary motif identified for ZIC1, 2 and 3 by Badis et al. (C_CAGC^A^/_G_GG). The experiments conducted here experimentally validate this motif for all ZIC proteins and show that functional binding of the ZIC Subclass A proteins can occur at this site in isolation or as part of a MTL. The Subclass B proteins can interact at this site but trans-activate it only when the site occurs within a MTL. The inability of the ZIC Subclass B proteins to elicit functional binding at an isolated, but multimerised, ZIC binding site may be because they lack Subclass A-specific trans-activator domains or because the DNA binding site is suboptimal for the Subclass B proteins. It would be interesting to determine the relative functional binding activity of each ZIC protein at the other ZIC1-3 binding sites identified by the protein binding microarray study of Badis et al.^20^ or to conduct a de novo search for an optimal Subclass B ZIC binding site.

To determine whether the ZIC Subclass A proteins contain trans-activation domains not found in the Subclass B proteins, regions of the Subclass A ZIC3 protein required for trans-activation were identified in a classic deletion mapping experiment. All variant proteins tested in the assays were shown to be stably produced and to localise to the cell nucleus. Previous experiments designed to detect homo- or hetero-meric ZIC complexes have not found evidence that ZIC proteins co-operate with themselves or other family members to elicit transcription^15^. Therefore, experiments with a multimerised binding site and overexpressed ZIC3 protein test the effect of protein domains on trans-activation free from confounding effects such as DNA accessibility and transcription factor co-operativity. When tested in this manner, only the newly identified Subclass A conserved domains SANC and SACC are necessary for trans-activation. Other previously identified evolutionarily conserved domains (the ZOC and ZFNC domains) behaved as transcriptional repressor domains. Additionally, two low complexity regions (often thought to be involved in trans-activation^39^ were either not required for trans-activation (the Alanine repeat region) or contributed a repressive influence (the Histidine repeat region). The ZIC3 trans-activation activity therefore localises to domains which are evolutionarily conserved amongst the Subclass A proteins, but lost from the Subclass B proteins. This is consistent with the inability of the Subclass B proteins to significantly trans-activate the isolated ZIC binding site, even when it is multimerised and the ZIC protein overexpressed, despite their ability to interact with the motif.

To determine how the ZIC proteins behave at loci bound during normal biology, a locus known to exhibit functional ZIC3 binding in embryonic stem (ES) cells^21^ was incorporated into the same vector as used for the multimerised binding site. The sequence, derived from the proximal promoter of the murine *Nanog* gene, contains binding sites for other transcription factors (OCT4, SOX2, ESRRB and KLF4) and was first identified as a multiple transcription factor locus^40^. ZIC3 is known to bind and stimulate *Nanog* transcription from this promoter region in conjunction with OCT4 and SOX2^21^. When incorporated into the ZIC trans-activation assay in HEK293T cells, ZIC3 activated this element at a similar level to that obtained in murine ES cells^21^ whereas the ZIC3-C365S variant protein did not activate it, demonstrating the activity is dependent upon interaction with the reporter DNA. Both Subclass B proteins were able to activate this element in HEK293T cells, indicating that the co-location of other transcription factors at a DNA sequence can enable the Subclass B proteins to promote transcription, despite their inability to do so at an isolated ZIC binding site.

This context dependent activity of the ZIC proteins was investigated further using the same ZIC3 domain deletion mapping approach as used with the multimerised binding site. Analysis revealed that many recognised ZIC domains exhibit different behaviour at the MTL element. For example, the SACC domain is required for maximal trans-activation at the multimerised binding site, but its removal does not significantly decrease transcription at the MTL. This suggests that interactions at the MTL can overcome the requirement for SACC as a trans-activator and that SACC is not required for these interactions. In contrast, removal of the SANC domain prevents some of the ZIC3 stimulated increase in trans-activation at the MTL indicative of a more robust requirement for this region. The three regions identified as necessary for transcriptional repression show altered activity at the MTL. Removal of ZOC or ZFNC does not elevate transcription at the MTL (as it does at the multimerised binding site) and removal of the Histidine repeat actually prevents some of the ZIC3 stimulated increase in trans-activation at the MTL. In each case, the repressive effect of these domains at the isolated ZIC3 binding site is prevented by interactions at the MTL. In a similar manner, the Alanine repeat domain (which plays no role in trans-activation per se) is necessary for optimal trans-activation at the MTL, indicative of a role in MTL interactions. This could account for the pathogenicity observed in human patients upon the expansion of this domain^41,42^. The experiments conducted here all use a loss-of-function (i.e. deletion) strategy that defines protein regions *necessary* for a given activity (in this case, trans-activation of reporter DNA). To determine whether any regions are *sufficient* for trans-activation, a gain-of-function assay is required (such as a GAL-4/UAS reporter assay). Furthermore, the experiments all rely on protein overexpression and it would be prudent to test the observations in a more physiological system.

The experiments reported here suggest that the overall transcriptional control ability of each ZIC protein is likely determined by their complement of domains that fall into three groups with respect to evolutionary conservation. First, a domain that is conserved amongst all family members (ZFNC), second, a domain whose type (i.e. low complexity region) is conserved in all ZIC proteins, but varies in number and composition and third, the Subclass A specific domains (ZOC, SANC and SACC). Some of these domains confer context dependent transcriptional behaviour and there is evidence that this is mediated by these regions acting as binding sites for protein partners^7,8^ and/or as targets of post-translational modifications^43^. Additionally, regions of low complexity sequence are also associated with the ability to bind multiple proteins and fold into different structures upon binding and are thought to be accessible to post translational modifications^44^. A picture therefore emerges of ZIC proteins with a ZFD flanked by regions of relative disorder. The flanking regions appear to participate in context specific interactions which ultimately determine the transcriptional outcome at a given ZRE. This flexibility is consistent with multiple proposed binding partners for the ZIC proteins^30,45–47^ and multiple biological roles of each family member. This work significantly advances our understanding of how ZIC proteins differentially control transcription. It identifies domains necessary for trans-activation that are absent from Subclass B proteins and other protein regions that control context dependent transcriptional control. The work will assist studies that assess likely pathogenicity of variant ZIC proteins found in the human genome or that seek to identify the context specific factors that influence functional transcription factor binding of the ZIC proteins.

## Methods

### Plasmids

The generation of V5-DEST, V5-ZIC2-wt, V5-ZIC3-wt, V5-ZIC5-wt, V5-ZIC3-katun, V5-ZIC3-C268X, V5-ZIC3-Q292X, V5-ZIC3-1507insTT and V5-ZIC3-K408X has been described previously^18^, as has V5-ZIC2-C371S^46^. The *APOE* reporter construct (B:*luc*^+^:APOE) was a gift from Francisco Zafra (Centro de Biología Molecular, Universidad Autonoma de Madrid, Spain)^17^. pGL4.20 (B:*luc2*) was purchased from Promega (Cat No. E675A). The remaining plasmids were generated as follows.

#### Expression constructs

Expression constructs were created using the Gateway Recombination Cloning Technology (Life Technologies). An entry clone containing the protein of interest coding DNA sequence (CDS) was generated using the pENTR3C vector (Life Technologies). Expression plasmids were created by transferring the CDS to pcDNA3.1/nV5-DEST expression vector (Life Technologies).

pENTR3C-ZIC1-wt: full-length ZIC1 CDS was PCR amplified from pcDNA-ZIC1-wt (a gift from Kathleen Millen, Seattle Children’s Hospital Research Foundation, USA) and cloned into the *EcoR*I sites of pENTR3C (Life Technologies) using the In-Fusion Dry-Down PCR Cloning System (Clontech).

pENTR3C-ZIC4-wt: full-length ZIC4 CDS was cloned by PCR amplifying the first 213 bp of ZIC4-wt CDS from genomic DNA (of HEK293T cells), while the 214-1005 portion of ZIC4 was PCR amplified from pcDNA-ZIC4-wt_214-1005 (a gift from Kathleen Millen, Seattle Children’s Hospital Research Foundation, USA). Full length ZIC4 CDS was created by joining these fragments in an overlap extension PCR and cloned into the *EcoR*I sites of pENTR3C (Life Technologies) using the In-Fusion Dry-Down PCR Cloning System (Clontech).

pENTR3C-ZIC5-C528S: Overlap extension PCR was used to introduce the C528S mutation within pENTR3C-ZIC5-WT to generate pENTR3C-ZIC5-C528S.

pENTR3C-ZIC1-C339S, pENTR3C-ZIC3-E155X, pENTR3C-ZIC3-C268S, pENTR3C-ZIC3-C302S, pENTR3C-ZIC3-C335S, pENTR3C-ZIC3-C365S, pENTR3C-ZIC3-C393S, and pENTR3C-ZIC4-C241S: mutations were created using the QuikChange Lightning Site-Directed Mutagenesis kit (Agilent Technologies; Cat. No. 210518) according to manufacturer’s instructions.

pENTR3C-ZIC3-ZOCdel, pENTR3C-ZIC3-ZFNCdel, pENTR3C-ZIC3-Ndel, pENTR3C-ZIC3-Aladel, pENTR3C-ZIC3-Hisdel, pENTR3C-ZIC3-SANCdel and pENTR3C-ZIC3-SACCdel: deletions mutants were made by Site-Directed Mutagenesis PCR. Mutagenesis PCRs were performed with the *PfuUltra* II Hotstart PCR Master Mix (Agilent Technologies; Cat. No. 600850) using 10 ng of pENTR3C-ZIC3-wt, according to manufacturer’s instructions. Following PCR, the amplified DNA was purified by ethanol precipitation and digested with *Dpn*I enzyme (NEB; Cat. No. R0176) for 2 h. DNA was again purified by ethanol precipitation and transformed into *E. coli*. Bacterial colonies were screened for the correct plasmid via DNA sequencing.

All ‘Entry’ clones (pENTR3C) were transferred to the pcDNA3.1/nV5-DEST expression vector via a Gateway LR Clonase reaction (Life Technologies) to generate V5-ZIC1-wt, V5-ZIC1-C339S, V5-ZIC3-E155X, V5-ZIC3-C268S, V5-ZIC3-C302S, V5-ZIC3-C335S, V5-ZIC3-C365S, V5-ZIC3-C393S, V5-ZIC4-C241S V5-ZIC3-ZOCdel, V5-ZIC3-ZFNC, V5-ZIC3-Ndel, V5-ZIC3-Aladel, V5-ZIC3-Hisdel, V5-ZIC3-SANCdel, V5-ZIC3-SACCdel, V5-ZIC4-wt, V5-ZIC4-C241S and V5-ZIC5-C528S.

#### Reporter constructs

B:*luc2*:β-globin, B:*luc2*:c-fos, B:*luc2*:TK: the β-globin promoter was PCR amplified from plasmid pKS:β-globin:lacZ^48^,the c-fos promoter was PCR amplified from TOPflash:c-fos plasmid (gift from Dr. Sabine Tejpar, Department of Oncology, Katholieke Universiteit Leuven, Belgium),the TK promoter was PCR amplified from TOPflash:TK plasmid (Upstate Biotechnology). Each amplicon was cloned into B:*luc2* using *Hind*III sites.

B:*luc2*:APOE, B:*luc2*:APOE:β-globin, B:*luc2*:Nanog, B:*luc2*:Nanog:β-globin, B:*luc2*:Foxd3 and B:*luc2*:Foxd3:β-globin: the *APOE* promoter region was PCR amplified from B:*luc*^+^:APOE; the *Nanog* promoter region was PCR amplified from mouse genomic DNA; the murine region equivalent to the chick *Foxd3* ZIC1 responsive enhancer^49^ was identified in RVista. The ZRE and flanking sequence (270 bp up- and down-stream) was PCR amplified from C57BL/6J genomic DNA. Each amplicon was cloned into B:*luc2* and B:*luc2*:β-globin using *Kpn*I and *Bgl*II sites.

B:*luc2*:Z3M2: the Z3M2 synthetic enhancer was constructed by using forward and reverse oligomers (Gene Link) that contained six repeats of Z3M2 (5′-CCC AGC GGG G-3′) separated by a 5-nucleotide spacer fragment (5′-TAG AA-3′). Oligomers were mixed in a 1:1 molar ratio and diluted to a concentration of 1 pmol/µL in oligo annealing buffer [10 mM Tris (pH = 8.0), 0.1 mM EDTA, 50 mM NaCl]. Annealing was carried out in PCR Thermal Cycler using the following program: 95 °C for 5 min, followed by 1 °C/min decrease in temperature for 70 min. Annealed oligomers were purified by ethanol precipitation and cloned into B:*luc2* using *Kpn*I and *Hind*III sites.

B:*luc2*:Z3M2:β-globin: The β-globin promoter (PCR amplified using Ark1510_F and Ark1507_R) was cloned into B:*luc2*:Z3M2 vector using *Hind*III sites.

B:*luc2*:Z3mtt:β-globin: the vector was obtained spontaneously during routine plasmid propagation. The Z3M2 site was mutated to 5′-CTA TCC CTG GGG GAG GGG GC-3′.

### Cell culture, transfection and western blotting

Cell culture, transfections and western blotting were performed as previously described^18^. In each experiment the nuclear fraction was used to detect expression of the transfected ZIC proteins using α-V5 (1:3,000 dilution; Life Technologies, R960-25) and to assess equal amounts of sample loading α-TATA binding protein (TBP) (1:2,000 dilution; Abcam, ab818) or α-LaminB1 (1:1,500; Abcam, ab16048).

### Luciferase assays

Luciferase assays were performed as previously described^18^ with the following modifications. Approximately 2 × 10^5^ cells, grown in flat bottom cell culture treated 12-well plates (Costar, CLS3513), were transfected with a total of 1.6 µg of DNA per well: 0.6 µg of reporter construct and either 1.0 µg of the expression construct or the negative control V5-DEST. 6 h post-transfection, cells were dissociated from the growth surface using 0.5 g/L trypsin (Life Technologies) and three replicate samples established by plating each transfection in triplicate on to a solid white tissue culture treated 96-well plate (Costar, CLS3917). 18 h after re-plating, cells in each well were lysed by incubation with 100 µL of a 1:1 dilution of luciferase substrate (ONE-Glo Luciferase Assay System, Promega) with Dulbecco’s Modified Eagle’s Medium (DMEM: Life Technologies) and the luminescence from each well measured using a TECAN Infinite M1000 Pro.

For each data point, the luciferase activity was normalised to the respective negative control V5-DEST luciferase value (such that the V5-DEST value becomes 1) to determine a relative luciferase activity: RLA. Following normalisation, the mean RLA and standard deviation (SD) was calculated from the three replicates (using Microsoft Excel). Each transfection was repeated three independent times (denoted by N) and the overall data set analysed as described under Statistical Analysis below.

### pIP-qPCR

HEK293T cells, grown in 100 mm TC dishes (Sigma-Aldrich; CLS430167) were transfected with 8 μg of the B:*luc2*:Z3M2 reporter construct or the negative control empty vector backbone pGL4.20 (B:luc2) and 16 μg of V5-ZIC-wt or V5-ZIC-variant construct. Six hours post-transfection, cells were dissociated from the growth surface using 0.5 g/L trypsin and plated in 150 mm TC dishes (Iwaki; 3030-150). 24 h post-transfection, media was removed and protein-DNA complexes cross-linked with 1.25% formaldehyde (w/v) (Sigma-Aldrich; F8775) at room temperature for 10 min. Cross-linking was terminated by the addition of 125 mM Glycine (Amresco; Cat. No. 0167) and cells washed thrice with ice-cold 1 × PBS. Cells were scraped in ice-cold 1 × PBS containing 0.02% Tween20 (Sigma-Aldrich; P7949) and pelleted via centrifugation. The pellet was resuspended in 1.8 mL of sonication buffer [50 mM Hepes (pH 7.9), 140 mM NaCl, 1 mM EDTA, 1% Triton X-100, 0.1% Na-deoxycholate, 0.1% SDS and protease inhibitors (AEBSF and PMSF)]. Cells were sonicated to obtain an average chromatin length of 500 bp using the Bioruptor (Diagenode) at 4 °C. To separate cellular debris sonicated samples were centrifuged (18,000g, 5 min, 4 °C).

Chromatin was pre-cleared with a 1:1 mixture of protein A (Novex; 10001D) and protein G (Novex; 10003D) Dynabeads for 2 h at 4 °C on a rotating platform. Pre-cleared chromatin was incubated with 7 μg of α-V5 antibody (Abcam; ab9116) in sonication buffer with protease inhibitors (rotating overnight, 4 °C). The following day, beads were washed at 4 °C once with ice-cold sonication buffer (with protease inhibitors) and thrice with sonication buffer containing 500 mM NaCl (with protease inhibitors), followed by one wash with ice-cold Lithium Chloride (LiCl) buffer (250 mM LiCl, 10 mM Tris–HCl (pH 8.0), 1 mM EDTA, 0.5% NP-40 and 0.5% Na-deoxycholate) and two washes with TE (10 mM Tris–HCl (pH 8.0), 1 mM EDTA). Beads were resuspended in 91.5 μL of TE and treated with 0.5 μg of RNase A (Thermo Fisher Scientific; Cat. No. EN0531) at 37 °C for 30 min. Cross-links were reversed by adding 5 μL of 10% SDS and 50 μg of Proteinase K (Thermo Fisher Scientific; Cat. No. EO0491) and incubation at 65 °C for 5 h. DNA was extracted using the AMPure purification system (according to manufacturer’s protocol).

qPCR was performed using 1 μL of a 1:10 dilution of input (10%) or pIP-enriched DNA, ImmoMix (Bioline; Cat. No. BIO-25020), 0.5 μM Ark1566_F (5′-CATTTCTCTGGCCTAACTGG-3′) and Ark1567_R (5′-AACAGTACCGGATTGCCAAG-3′) primers, and SYBR Green dye in a 10 μL volume. PCRs were run using the StepOne Real-time PCR machine (Applied Biosystems) with the following PCR cycling conditions: 95 °C for 5 min, followed by 40 cycles of 95 °C for 15 s, 55 °C for 20 s and 72 °C for 30 s and a melt curve stage that included 95 °C for 15 s, 55 °C for 1 min and 0.3 °C increase in temperature every 15 s until it reached 95 °C. Data was analysed using the StepOne Software (v2.3). The amount of target-specific DNA precipitated was determined relative to the amount of non-immunoprecipitated (input) DNA, using the percent input method as outlined on: https://www.thermofisher.com/au/en/home/life-science/epigenetics-noncoding-rna-research/chromatin-remodeling/chromatin-immunoprecipitation-chip/chip-analysis.html.

### Statistical analysis

Luciferase assays: A two-way analysis of variance (ANOVA) on raw luminescence data pooled from three independent repeat transfections was performed via Genstat. The mean and a single experimental Standard Error of the Mean (SEM) was calculated for all treatment groups. The same operation performed to calculate the RLA was used to calculate a normalised SEM value i.e. normalised SEM values for RLA were calculated by dividing the SEM of raw luminescence values by the mean luminescence value of V5-DEST from three experimental repeats. A Post Hoc test using the Bonferroni correction method (α = 0.01) was performed to identify treatment groups that were significantly different. When the difference between means of two treatment groups was larger than the computed Least Significant Difference (LSD), the treatments were considered significantly different. The software assigned a unique letter (a, b, c, d, e…) to the treatment group that was significantly different from all other treatments. When the difference between means of two treatment groups was less than the LSD, they were assigned the same letter indicating no significant difference.

pIP-qPCR: Excel was used to perform a *t*-Test (one-tailed) on the mean percent input values.

## Supplementary information

Supplementary Figures.
