# Supplementary material for: Systematized reporter assays reveal ZIC protein regulatory abilities are Subclass-specific and dependent upon transcription factor binding site context
Source: Sci Rep. 2020 Aug 4;10:13130. doi: 10.1038/s41598-020-69917-9 (PMC7403390; doi:10.1038/s41598-020-69917-9)
Supplement: Supplementary file 1 — Supplementary Figures. [file 41598_2020_69917_MOESM1_ESM.pdf]

Supplementary Information

Systematized reporter assays reveal ZIC protein regulatory abilities are Subclass-specific and dependent upon transcription factor binding site context

Jehangir N. Ahmed[1], Koula E. M. Diamand[1], Helen M. Bellchambers[1,2] and Ruth M. Arkell[1,*]

1: Early Mammalian Development Laboratory, John Curtin School of Medical Research, The Australian National University, Canberra, ACT 2601, Australia

2: Present address: Department of Pediatrics, Indiana University School of Medicine, Indianapolis, IN, USA

*: Author for Correspondence

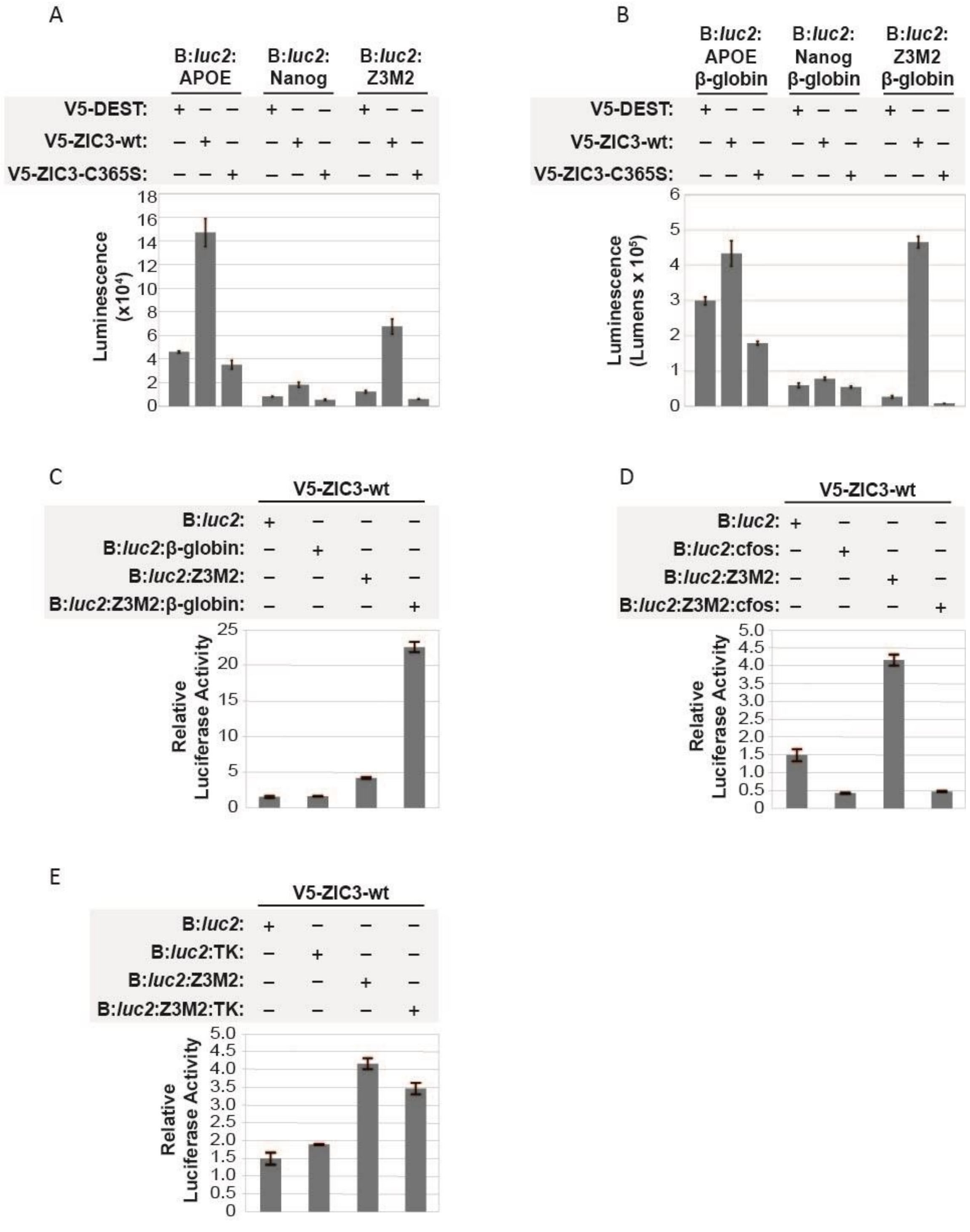

Figure S1: Analysis of reporter constructs suitable for ZIC transactivation studies. (A, B) This supporting data corresponds to main Figure 1H and 1J. Raw luciferase values from one representative experiment including V5-DEST is shown. Error bars represent SD between three internal replicates. **(C, D, E)** Combining a minimal promoter with the Z3M2 synthetic enhancer. HEK293T cells were transfected with the reporter plasmids shown and V5-DEST or V5-ZIC3-wt. Data shown represents RLA via V5-ZIC3-wt for each reporter (with reference to V5-DEST). Errors bars represent SD between internal repeats from one representative experiment.

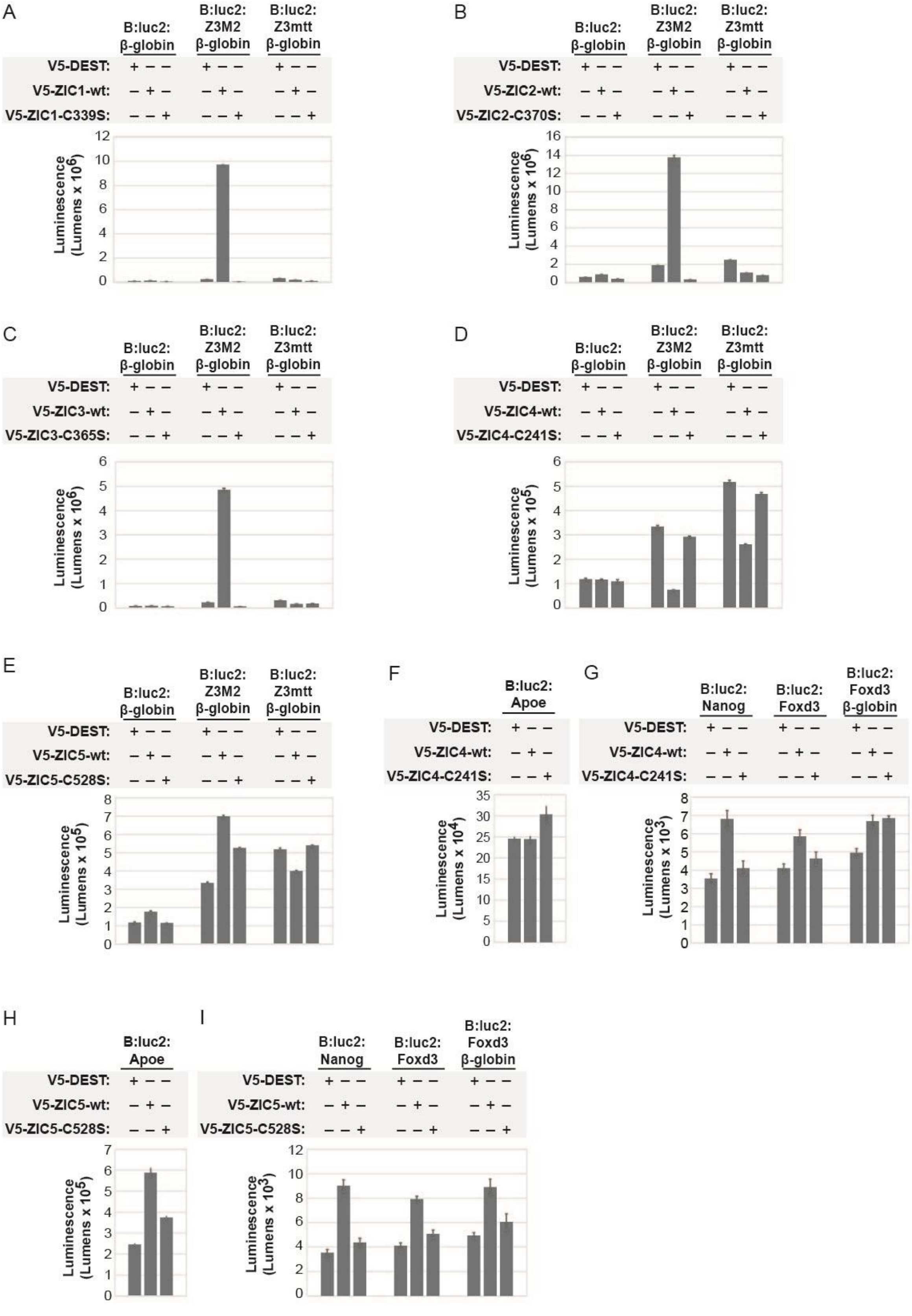

Figure S2: Trans-activation spectrum of ZIC proteins with inclusion of V5-DEST. This supporting data corresponds to all panels of Figure 2. Raw luciferase values from one representative experiment including V5-DEST is shown. Error bars represent SD between three internal replicates.

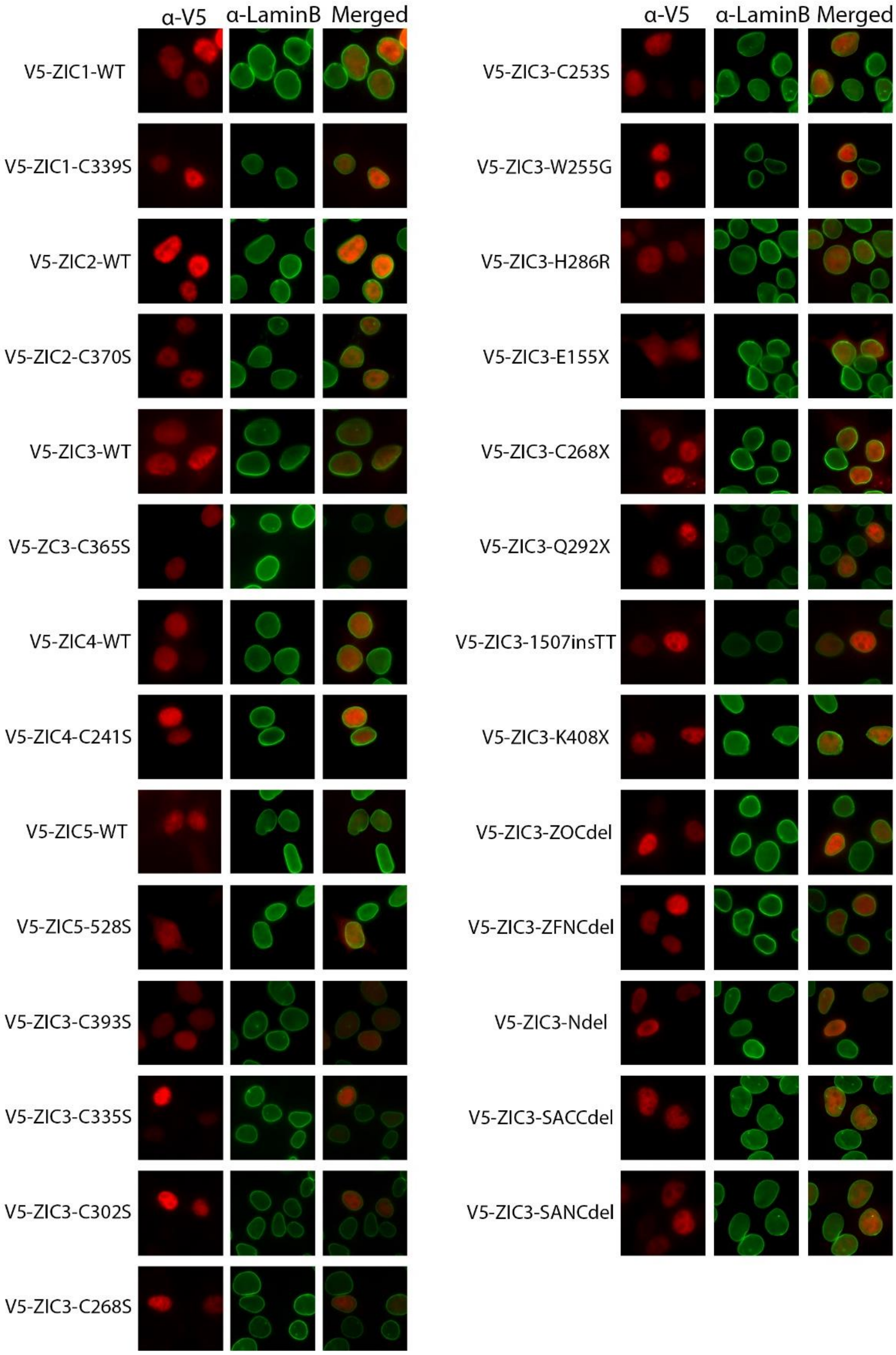

Figure S3: Subcellular distribution of ZIC3 variants. V5-tagged ZIC3 expression constructs containing the relevant mutations were transfected into HEK293T cells. 24 hours post-transfection cells were prepared for immunofluorescence microscopy and stained with α-V5 (to detect the transfected protein) and α-LaminB1 (to mark the nuclear boundary).

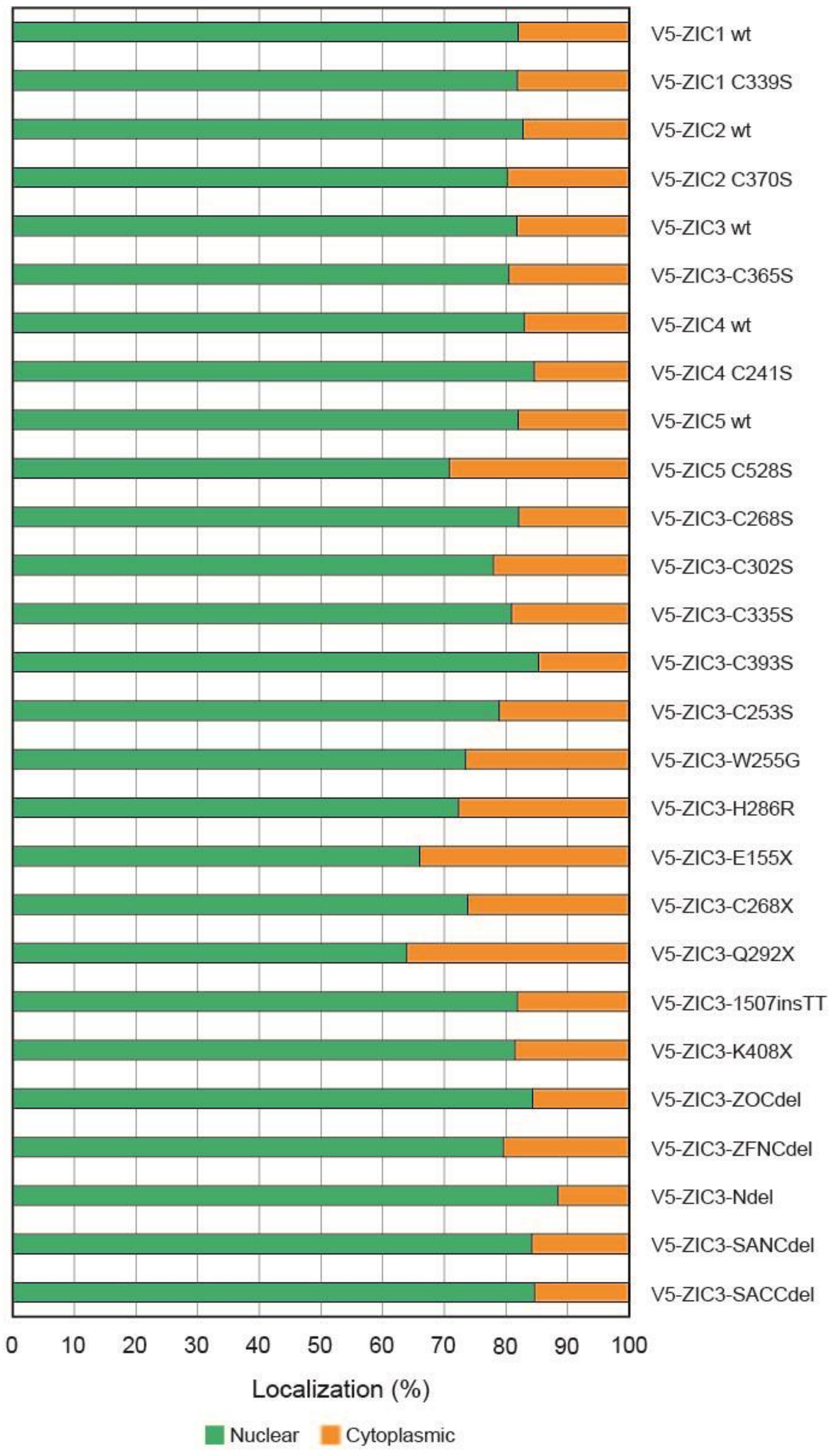

Figure S4: Quantification of the subcellular distribution of ZIC3 variants. Subcellular localisation of transfected V5-ZIC3 protein (Figure S3) was quantified using ImageJ software**.** Green and orange regions represent the nuclear and cytoplasmic compartments, respectively.
